# Characterization of Immunological Responses to *Borrelia* Immunogenic Protein A (BipA), a Species-Specific Antigen for North American Tick-Borne Relapsing Fever

**DOI:** 10.1128/spectrum.01722-21

**Published:** 2022-05-17

**Authors:** Michael W. Curtis, Aparna Krishnavajhala, Alexander R. Kneubehl, Monica E. Embers, Jenna R. Gettings, Michael J. Yabsley, Job E. Lopez

**Affiliations:** a Department of Pediatrics, National School of Tropical Medicine, Baylor College of Medicine, Houston, Texas, USA; b Department of Molecular Virology and Microbiology, Baylor College of Medicine, Houston, Texas, USA; c Division of Immunology, Tulane National Primate Research Center, Tulane University Health Sciences, Covington, Louisiana, USA; d Southeastern Cooperative Wildlife Disease Study, Department of Population Health, College of Veterinary Medicine, University of Georgia, Athens, Georgia, USA; e Warnell School of Forestry and Natural Resources, University of Georgia, Athens, Georgia, USA; University of Cincinnati

**Keywords:** *Borrelia hermsii*, *Borrelia parkeri*, *Borrelia turicatae*

## Abstract

Tick-borne relapsing fever (TBRF) is a neglected vector-borne bacterial disease distributed worldwide. Borrelia turicatae, Borrelia parkeri, and Borrelia hermsii are three argasid-borne TBRF species previously implicated in human disease in North America. TBRF is likely underdiagnosed due to its nonspecific symptoms and poorly developed diagnostic tests. Studies suggest that the *Borrelia* immunogenic protein A (BipA) is specific to TBRF *Borrelia* but heterogenic between species. In this study, we hypothesized that antibody responses generated to BipA are specific to the North American TBRF species infecting a given animal. To test this, we characterized the expression and localization of native BipA in North American species of TBRF *Borrelia*. We also infected mice by needle inoculation or tick bite with *B. turicatae,* B. hermsii, or *B. parkeri* and evaluated serum sample reactivity to recombinant BipA (rBipA) that was produced from each species. Furthermore, serum samples from nonhuman primates and domestic dogs experimentally infected with *B. turicatae* were assessed. Lastly, we tested human Lyme disease (LD) serum samples to determine potential cross-reactivity to rBipA generated from *B. turicatae*, *B. parkeri*, and B. hermsii. Our findings indicate that rBipA has the potential to distinguish between infections of LD- and TBRF-causing spirochetes and that antibody responses were more robust toward the *Borrelia* species causing infection. This work further supports that rBipA can likely distinguish between *B. turicatae*, B. hermsii, and *B. parkeri* infections in mice, canines, and nonhuman primates.

**IMPORTANCE** *Borrelia* species transmitted by soft or hard ticks cause tick-borne relapsing fever (TBRF). This is a debilitating disease distributed worldwide but is likely underdiagnosed or misdiagnosed as Lyme disease due to poorly developed diagnostic tests. Borrelia turicatae, Borrelia parkeri, and Borrelia hermsii are three TBRF species previously implicated in human disease in North America. Commonly used diagnostic methods do not identify the species causing infection. In this study, we evaluated the potential of recombinant *Borrelia* immunogenic protein A (rBipA) as a diagnostic antigen capable of distinguishing between infections of TBRF *Borrelia* species. We show that serum from mice, canines, and nonhuman primates infected with *B. turicatae*, *B. parkeri*, or B. hermsii react more strongly to the rBipA from the species causing infection. Furthermore, sera from Lyme disease patients failed to cross-react with our rBipA proteins, indicating the potential to use rBipA as a species-specific diagnostic antigen for TBRF.

## INTRODUCTION

Tick-borne relapsing fever (TBRF) is a global yet neglected disease caused by at least 15 different pathogenic species from the *Borrelia* genus (1). TBRF *Borrelia* are primarily transmitted to susceptible hosts by argasid (soft) ticks. If untreated, the spirochetes establish an infection in the blood, and the disease manifests with recurring episodes of fever, headache, myalgia, chills, nausea, neurological complications, miscarriage, and potential death (2–6). Borrelia hermsii and Borrelia turicatae cause the majority of human TBRF cases in North America (7–11), while Borrelia parkeri, Borrelia mazzottii, and “*Candidatus* Borrelia johnsonii” have also been implicated in human disease (12–16).

The burden of TBRF in humans and domestic animals is unclear because it is likely under- and misdiagnosed due to its nonspecific clinical manifestations and poorly developed diagnostic tests. For example, TBRF has been misdiagnosed as viral infections or malaria due to the presentation of high fevers associated with additional nonspecific symptoms (17, 18). Since TBRF spirochetes attain high densities in the blood compared to those of Lyme disease (LD)-causing pathogens, an often used method to diagnose TBRF is through the direct observation of bacteria in a blood smear. However, this method has a low sensitivity, with the limit of detection between 10^3^ and 10^4^ cells per mL of blood (19, 20). Additionally, it may become obsolete due to the continued discovery of novel pathogenic spirochetes, such as Borrelia mayonii, which is an LD-causing spirochete capable of attaining high levels of spirochetemia (21). Amplification of nucleic acid is more sensitive than microscopic observation (22–24), and has the added ability to define the species of infection (18, 24). However, its ability to detect neuroborreliosis caused by TBRF remains challenging (25). Furthermore, PCR analysis has limited use in retrospective diagnoses and epidemiologic surveillance. Further hindering accurate TBRF diagnosis is the lack of commercially available serologic tests and cross-reactivity of TBRF-positive serum samples to antigens from LD-causing spirochetes (26–29).

To address the cross-reactivity observed between serum samples from patients or wild animals infected with TBRF- or LD-causing spirochetes, three antigens have been identified and characterized. Glycerophosphodiester phosphodiesterase (GlpQ), factor H binding protein A (FhbA), and *Borrelia* immunogenic protein A (BipA) are TBRF antigens capable of differentiating between the two diseases (30–32). An analysis with BipA identified low amino acid homology between B. hermsii and *B. turicatae* homologues (33). Moreover, studies determined that in mice and a domestic dogs recombinant BipA (rBipA) could be used in serological assays to differentiate between the two infections (34).

In this study, we further assessed the diagnostic capabilities of rBipA between North American species of TBRF spirochetes. We hypothesize that due to the heterogeneity of BipA between TBRF *Borrelia* species, recombinant proteins from B. hermsii, *B. parkeri*, and *B. turicatae* could differentiate between infections from a given species. To evaluate BipA as a diagnostic antigen, the production and surface localization of the protein was determined for *B. turicatae* and *B. parkeri*. To test the antigenicity of rBipA, we evaluated murine serum samples that were generated by needle-inoculating animals or infecting them by tick bite. We also assessed domestic dogs and rhesus macaque serum samples generated from previous work (27, 35). A cohort of human LD-positive serum samples was also tested (36). We evaluated their cross-reactivity to rBipA generated from *B. turicatae*, *B. parkeri*, and B. hermsii. Collectively, our findings indicate the potential of rBipA to discriminate between infections of LD- and TBRF-causing spirochetes and to be a species-specific diagnostic antigen for North American TBRF spirochetes.

## RESULTS

### Sequence analysis of BipA.

**FIG 1:**
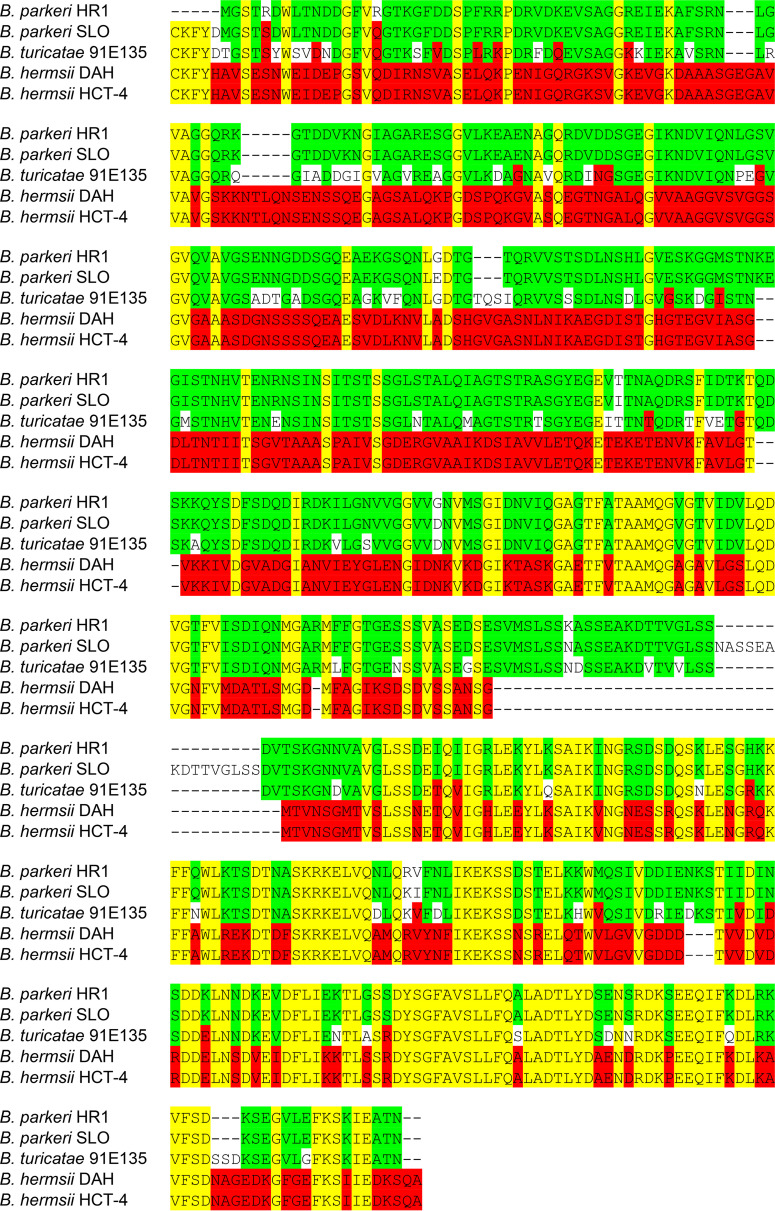
Amino acid alignment of BipA from *B. turicatae*, *B. parkeri*, and B. hermsii strains. ClustalW alignment of the amino acid sequence of the mature BipA proteins from *B. turicatae* 91E135 (YP_008145285.1), *B. parkeri* HR1 (AHF45615.1), *B. parkeri* SLO (MW589542), B. hermsii DAH (ACS27065.1), and B. hermsii HCT-4 (MW589543). Yellow highlights indicate amino acid conservation between all five TBRF strains. Green highlights indicate additional amino acid conservation between *B. parkeri* HR1, *B. parkeri* SLO, and *B. turicatae* 91E135. Red highlights indicate additional amino acid conservation between B. hermsii DAH, B. hermsii HCT-4, and *B. turicatae* 91E135.

A challenge encountered in this study was the differences in strains used to produce rBipA compared to the ones used to infect mice. For example, rBipA was originally produced from the *B. parkeri* HR1 and B. hermsii DAH isolates because these genomes were available on GenBank and the *bipA* nucleotide sequences were identified. However, the laboratory strains used to infect mice and to generate protein lysates for immunoblots were *B. parkeri* SLO and B. hermsii HCT-4 because they were available in our laboratory. Consequently, we sequenced *B. parkeri* SLO and B. hermsii HCT-4 genomes, and BipA amino acid alignments were performed to determine sequence identity to HR1 and DAH. The ClustalW alignment showed 96% BipA amino acid identity between *B. parkeri* SLO and HR1 (Fig. 1). In the *B. parkeri* HR1 BipA sequence submitted to GenBank (AHF45615.1) a signal peptide was absent; however, our sequencing of *B. parkeri* SLO BipA (MW589542) revealed a consensus signal peptide. Amino acid alignments also indicated there was 100% sequence identity between B. hermsii DAH and HCT-4 (Fig. 1). Furthermore, there was 79 to 81% interspecies amino acid identity between *B. turicatae* and both *B. parkeri* strains and 39% amino acid identity between *B. turicatae* and both strains of B. hermsii (Fig. 1). Given the high degree of intraspecies amino acid identity of BipA in *B. parkeri* and B. hermsii strains, we reasoned that using different isolates to infect mice would not adversely impact our findings.

### Validation of rabbit anti-*B. turicatae* rBipA serum.

**FIG 2:**
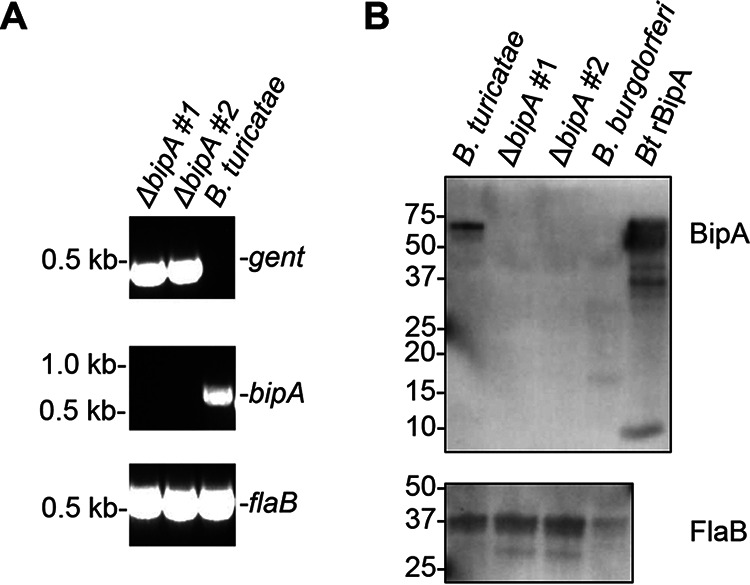
Inactivation of *bipA* and assessment of BipA protein production in wild-type *B. turicatae* and mutants (Δ*bipA* clones). (A) PCR amplification using internal primers located within *gent* (top panel), *bipA* (middle panel), or *flaB* (bottom panel). Molecular sizes are shown to the left of each gel. (B) Wild-type *B. turicatae*, Δ*bipA* mutants, and B. burgdorferi were probed with polyclonal rabbit anti-*B. turicatae* rBipA (top panel) and polyclonal chicken anti-*B. turicatae* rFlaB serum (bottom panel). The molecular weights (kDa) are shown to the left of each membrane.

Since rabbit anti-*B. turicatae* rBipA serum was commercially produced, we validated the sample’s specificity using wild type *B. turicatae*, two *B. turicatae* Δ*bipA* mutants, and wild-type *Borreliella* (*Borrelia*) *burgdorferi* B31 A3. The *bipA* gene was inactivated in *B. turicatae* through homologous recombination with a vector containing the gentamicin resistance cassette (P_flg_-gent) flanked by 1,000 bp upstream and downstream of the *bipA* (see Fig. S1 in the supplemental material). PCR confirmed the inactivation of *bipA* in two *B. turicatae* Δ*bipA* clones (Fig. 2A). Immunoblots using two Δ*bipA* clones validated the specificity of the rabbit anti-*B. turicatae* rBipA serum (Fig. 2B). The rabbit serum detected an ~60 kDa band in protein lysate of wild-type *B. turicatae* and rBipA, but reactivity to protein lysates of the Δ*bipA* mutants and B. burgdorferi was absent (Fig. 2B). Chicken anti-*B. turicatae* flagellin (FlaB) polyclonal antibodies confirmed that similar amounts of protein lysates from wild-type *B. turicatae* and Δ*bipA* mutants were used in the assay (Fig. 2B).

### Production and localization of BipA in North American TBRF *Borrelia*.

**FIG 3:**
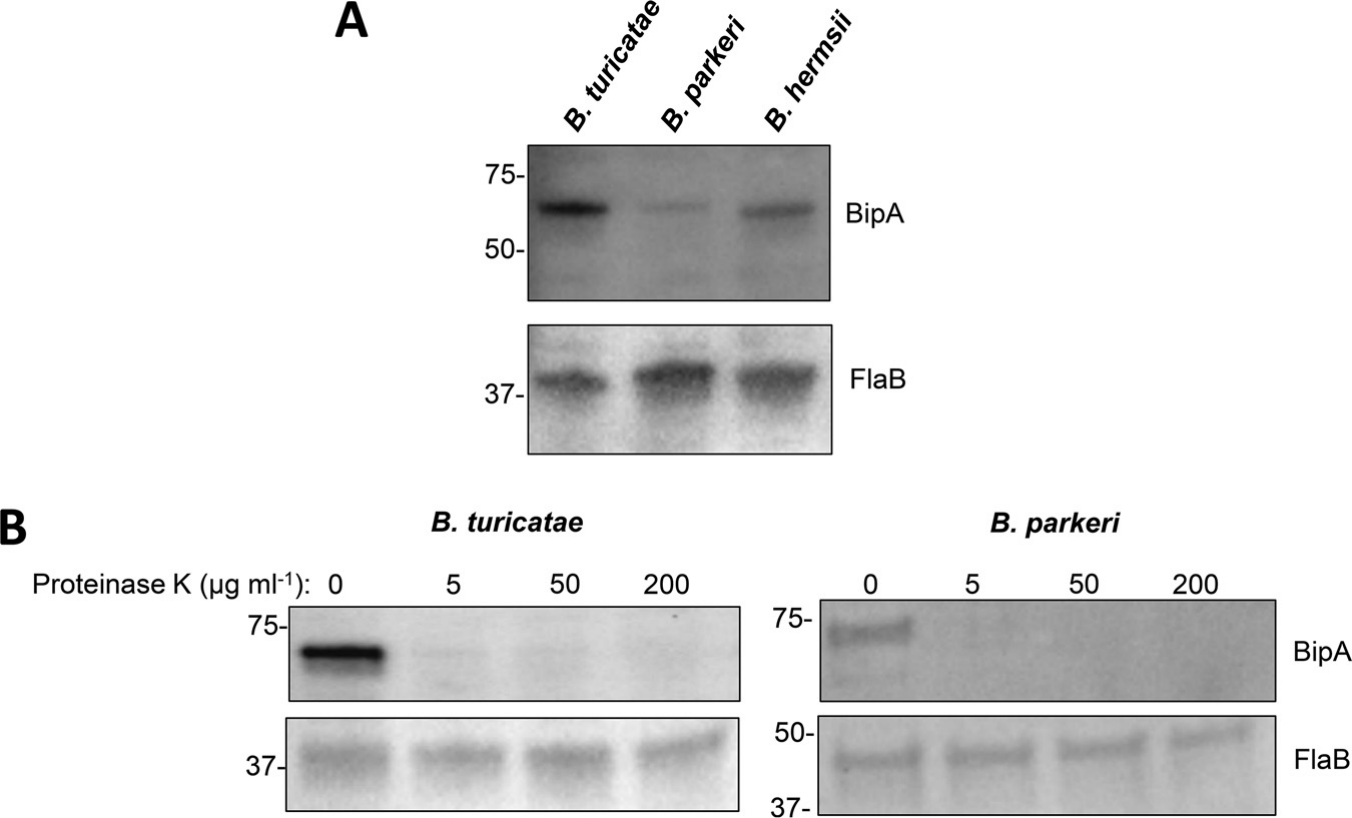
BipA production and surface localization. (A) Protein lysates of *B. turicatae*, *B. parkeri*, and B. hermsii were probed with rabbit polyclonal anti-*Bt*-BipA antibodies (top panel) or chicken anti-*B. turicatae* recombinant flagellin (rFlaB) (bottom panel). (B) Protein lysates of *B. turicatae* and *B. parkeri*, which were incubated with increasing concentrations of proteinase K for 15 min at room temperature, were probed with antibodies generated against BipA (top panels) and FlaB (bottom panels). Blots are representative of two independent experiments. The molecular weights (represented in kDa) are shown to the left of the blots.

A series of immunoblotting assays were performed to detect BipA and determine its surface localization in North American TBRF *Borrelia* species. Polyclonal rabbit anti-*B. turicatae* rBipA serum detected the ~60-kDa protein in *B. turicatae*, *B. parkeri*, and B. hermsii (Fig. 3A, top panel). Probing for FlaB indicated that similar amounts of protein lysate were electrophoresed in the gels (Fig. 3A, bottom panel). This was the first confirmation of the production of BipA in *B. parkeri*. These results also indicated that rabbit anti-BipA polyclonal serum bound to conserved epitopes in the three species tested.

Proteinase K assays were also performed to determine if BipA is exposed on the surface of *B. turicatae* and *B. parkeri* (Fig. 3B). We did not assess B. hermsii, because the surface localization of BipA in this species was previously demonstrated through proteinase K assays (37). Since proteinase K is a serine protease incapable of penetrating the outer membrane of *Borrelia* (38), detection of the periplasmic FlaB was performed to ensure the integrity of the bacterial outer membrane (35, 38–40). The incubation of cells with increasing concentrations of proteinase K (0, 5, 50, and 200 μg mL^−1^) for 15 min resulted in the degradation of BipA, while FlaB remained intact (Fig. 3B). These results indicated that BipA is exposed on the surface of *B. turicatae* and *B. parkeri*.

### Generation of serum samples.

We used murine, canine, and nonhuman primate (NHP) serum samples from previous work (27, 34, 35, 41) and generated additional murine serum samples by infecting animals by tick transmission or needle inoculation (Table 1). Within 5 to 7 days after feeding ticks on mice, we visualized *B. turicatae* 91E135 or *B. parkeri* SLO in the blood. Similarly, mice became infected with B. hermsii HCT-4 following needle inoculation. Animals were exsanguinated 4 weeks after infection, and seroconversion was assessed. One mouse inoculated with B. hermsii HCT-4 was exsanguinated 17 days postinoculation due to signs of morbidity. Seroconversion was confirmed positive by Western blotting using *B. turicatae* 91E135, *B. parkeri* SLO, or B. hermsii HCT-4 protein lysates.

**TABLE 1:** Serum samples used in the study^*a*^

RF *Borrelia*	Route of infection	Animal	No. of animals	Source or reference
*B. turicatae*	Tick	Mouse	19	41, this study
*B. turicatae*	Tick, needle inoculation	Canine	6	27, 34
*B. turicatae*	Tick	NHP	3	35
B. hermsii	Needle inoculation	Mouse	16	This study
*B. parkeri*	Tick	Mouse	20	This study

a RF, relapsing fever; NHP, nonhuman primate.

### Immunoblotting to determine serum reactivity from infected mice against rBipA.

**FIG 4:**
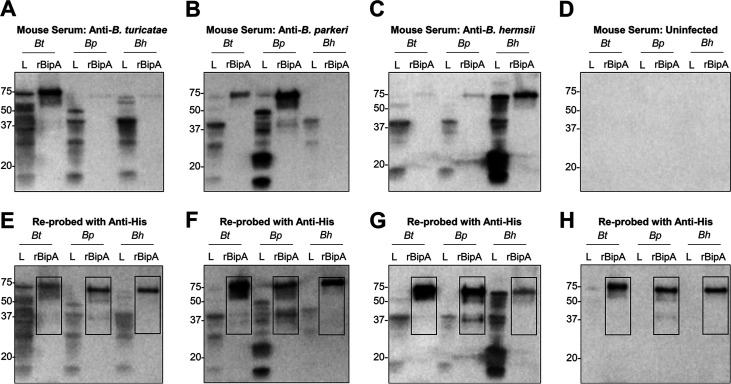
Mouse antibody responses to rBipA from North American TBRF species. *B. turicatae* (*Bt*), *B. parkeri* (*Bp*), and B. hermsii (*Bh*) protein lysates (L) and rBipA from each TBRF *Borrelia* species were electrophoresed and transferred to membranes. (A to C) Shown are serum samples from mice infected with *B. turicatae* (A), *B. parkeri* (B), or B. hermsii (C). (D) Serum from an uninfected mouse was used as a negative control. (E to H) Immunoblots were also reprobed with a monoclonal antibody generated against the 10× histidine tag on each recombinant protein. Blots are representative of three mice infected with *B. turicatae*, *B. parkeri*, or B. hermsii. Molecular weights are shown in kilodaltons to the left of each immunoblot.

Serum samples from mice infected with *B. turicatae*, *B. parkeri*, or B. hermsii were used to evaluate reactivity toward rBipA from each TBRF *Borrelia* species (*Bt-*, *Bp-*, and *Bh-*rBipA). Serum from a mouse infected with *B. turicatae* detected antigens in *B. turicatae*, *B. parkeri*, and B. hermsii protein lysates (Fig. 4A). The serum sample also detected *Bt-*rBipA at 17-fold and 20-fold higher optical density than *Bp*-rBipA and *Bh*-rBipA, respectively (Fig. 4A). A serum sample from a mouse infected with *B. parkeri* detected antigens in *B. turicatae*, *B. parkeri*, and B. hermsii protein lysates (Fig. 4B). *Bp-*rBipA was detected at 5-fold and 100-fold higher optical density than *Bt*-rBipA and *Bh*-rBipA, respectively (Fig. 4B). Also, a serum sample from a mouse infected with B. hermsii detected antigens in *B. turicatae*, *B. parkeri*, and B. hermsii protein lysates (Fig. 4C). The serum sample also detected *Bh-*rBipA at 20-fold and 6-fold higher optical density than *Bt*-rBipA and *Bp*-rBipA, respectively (Fig. 4C). Serum from an uninfected mouse did not react to *Borrelia* protein lysates or rBipAs (Fig. 4D). Blots reprobed with a monoclonal anti-polyhistidine antibody demonstrated the presence of recombinant proteins (Fig. 4E to H). The blot initially probed with uninfected mouse serum was reprobed with a monoclonal anti-polyhistidine antibody, which showed the relative abundance of each rBipA (Fig. 4H).

### Detection of antibody responses to BipA by ELISA.

**FIG 5:**
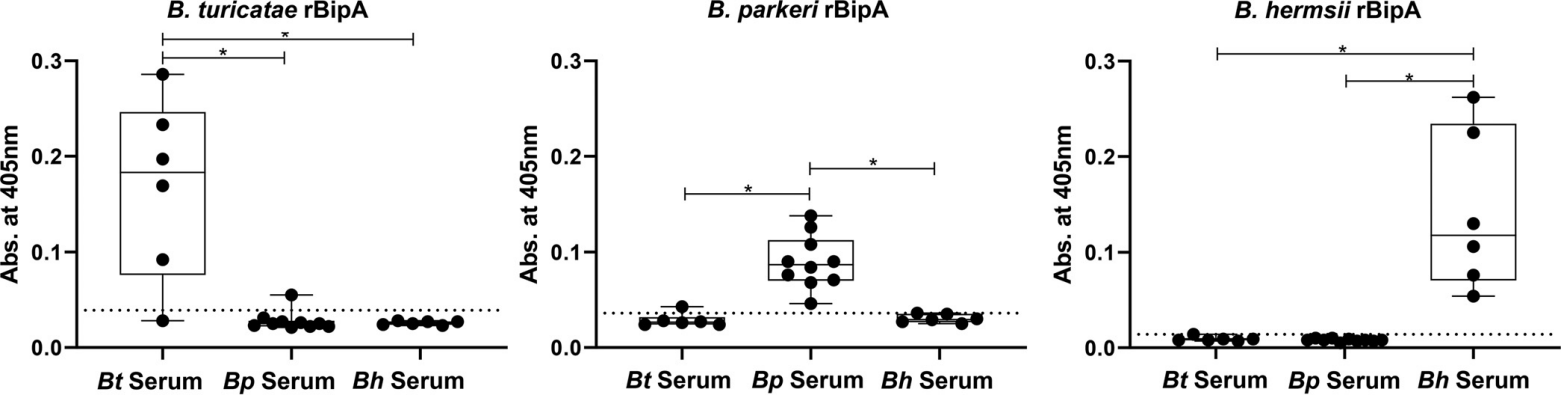
Species-specific immune responses toward rBipA. Absorbances using serum from mice infected with *B. turicatae*, *B. parkeri*, or B. hermsii binding to *Bt*-rBipA, *Bp*-rBipA, and *Bh*-rBipA are shown in box and whisker graphs. The dashed line depicts the threshold of a positive result for each rBipA (mean plus three times the standard deviation of uninfected controls). Statistical significance is shown with an asterisk (*, *P ≤ *0.01). Graphs are representative of ELISAs repeated twice.

In enzyme-linked immunsorbent assays (ELISAs), sera from mice infected with *B. turicatae*, *B. parkeri*, or B. hermsii were used to detect *Bt*-rBipA, *Bp-*rBipA, and *Bh*-rBipA. In total, 19 *B. turicatae*, 20 *B. parkeri*, and 16 B. hermsii serum samples were assessed, and each ELISA was repeated twice. Shown are results from a single ELISA plate, which are representative of the other assays (Fig. 5). Serum samples from mice infected with *B. turicatae* had significantly higher absorbances for *Bt*-rBipA than *Bp*-rBipA and *Bh*-rBipA (Fig. 5, left panel). Similarly, sera from mice infected with *B. parkeri* or B. hermsii had significantly higher absorbances for the rBipA of the infecting species (Fig. 5, middle and right panels). We observed stronger binding toward the BipA from the infecting species, regardless of whether a mouse was infected by tick bite or needle inoculation.

Sensitivity and specificity values were also determined for the rBipAs that were evaluated, and a given sample was considered positive if the absorbance was greater than the mean of the uninfected serum samples plus three times the standard deviation. The use of *Bt*-rBipA, *Bp*-rBipA, and *Bh*-rBipA to distinguish *B. turicatae*, *B. parkeri*, or B. hermsii infection in mice resulted in specificities of ≥97% and sensitivities of ≥82% (Table 2). Even though seven samples tested positive for multiple rBipAs, all had higher titers toward the rBipA from the infecting species compared to rBipA from other species (Fig. S2). Taken together, these data indicated that serum from TBRF-positive mice reacted more robustly toward the rBipA from the *Borrelia* species causing infection.

**TABLE 2:** Sensitivity and specificity of rBipA ELISA serology test

Organism	Sensitivity (%)	Specificity (%)
*B. turicatae* rBipA	93	98
*B. parkeri* rBipA	82	97
B. hermsii rBipA	83	97

### Evaluation of serological responses to *B. turicatae* rBipA in cohorts of infected canines and nonhuman primates.

To assess if the species-specific immunological responses toward BipA occur in other mammals, serum samples from canines and nonhuman primates experimentally infected with *B. turicatae* were used (27, 34, 35). Serum samples from domestic dogs infected with *B. turicatae* for 54 days had at least 4-fold higher antibody titers toward *Bt*-rBipA than *Bp*-rBipA and 8-fold higher titers than *Bh*-rBipA (Table 3). Serum collected 85 days post-*B. turicatae* infection from nonhuman primates had at least 2-fold higher antibody titers toward *Bt*-rBipA than *Bp*-rBipA and at least 8-fold higher titers than *Bh*-rBipA (Table 3). These data suggest that BipA can differentiate between the species of relapsing fever *Borrelia* causing the infection in higher-order mammals.

**TABLE 3:** IgG titers of Borrelia turicatae-infected domestic dogs and nonhuman primates (NHP)

Serum sample	IgG titers of:		
	*B. turicatae* rBipA	*B. parkeri* rBipA	B. hermsii rBipA
Canine serum 1	1,600	400	200
Canine serum 2	1,600	200	<200
Canine serum 3	1,600	400	<200
Canine serum 4	1,600	200	<200
Canine serum 5	1,600	400	200
Canine serum 6	800	<200	<200
NHP serum 1	3,200	1,600	800
NHP serum 2	6,400	800	400
NHP serum 3	3,200	1,600	400

### Evaluation of sera reactivity against rBipA from LD patients.

**FIG 6:**
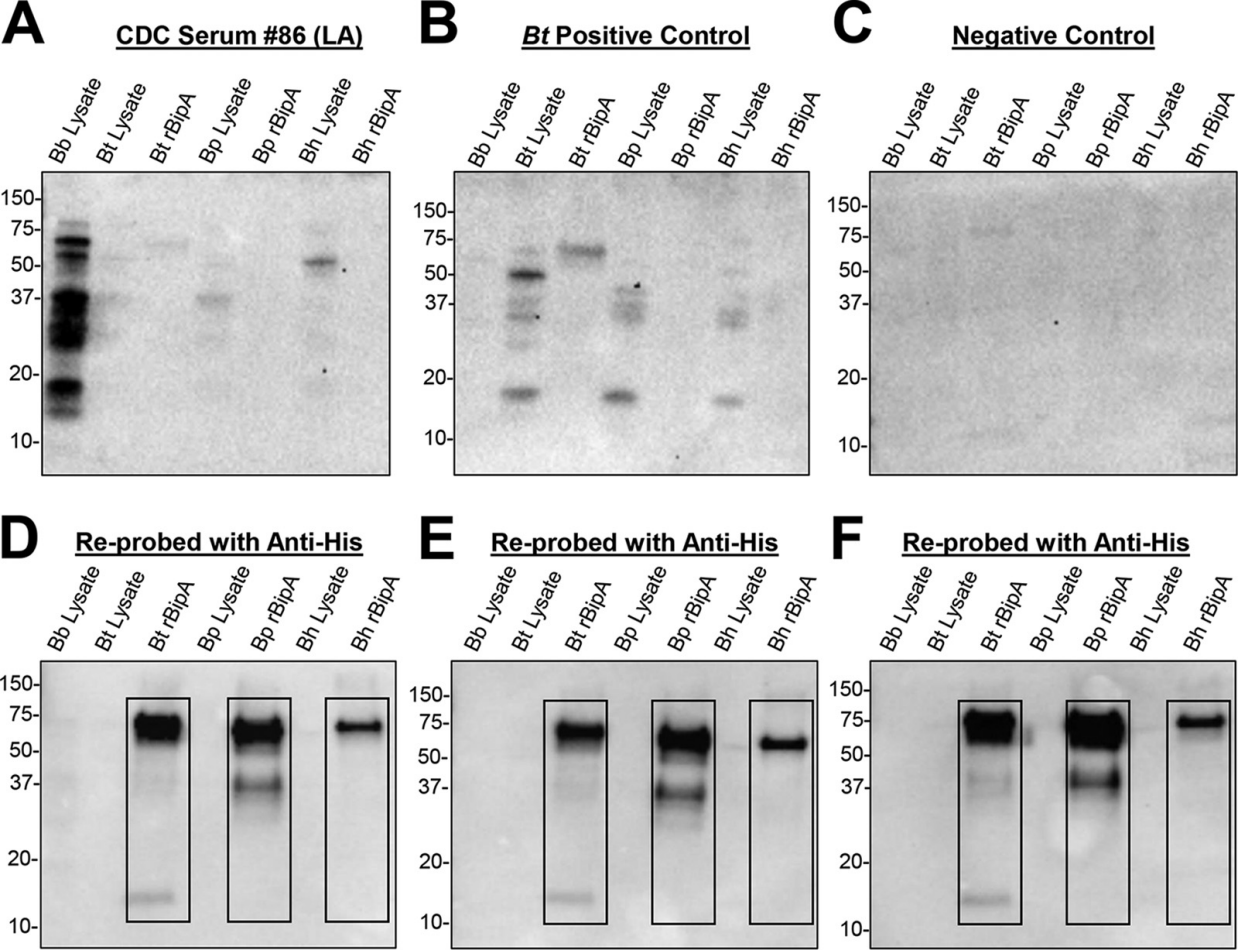
Reactivity of serum from LD patients toward rBipA from North American TBRF species. Protein lysates from B. burgdorferi (Bb), *B. turicatae* (Bt), *B. parkeri* (Bp), B. hermsii (Bh), and rBipA from each of the TBRF *Borrelia* species were used for immunoblots. (A) Representative blots from the Lyme arthritis patient that was positive to rBipA by ELISA. (B and C) A serum sample from a TBRF patient is shown as a positive control (B), and a negative human serum sample was used for a negative control (C). (D to F) Immunoblots were also reprobed with a monoclonal antibody generated against the 10× histidine tag on each recombinant protein. Molecular weights are shown in kilodaltons to the left of each immunoblot.

Serum samples from LD patients were evaluated for reactivity toward rBipAs from *B. turicatae*, *B. parkeri*, and B. hermsii. In an ELISA, a single serum sample from a Lyme arthritis patient tested positive for *Bt*-rBipA and *Bp*-rBipA at a 1:200 dilution (Table 4). In immunoblots, the ELISA positive serum sample was negative at a 1:200 dilution (Fig. 6A). Moreover, none of the remaining LD patient serum samples reacted to *Bt*-rBipA, *Bp*-rBipA, or *Bh*-rBipA at a dilution of 1:200. Immunoblots were also probed with a serum sample from a *B. turicatae*-infected patient and negative-control serum sample (Fig. 6B and C). Immunoblots were reprobed with an anti-polyhistidine monoclonal antibody to confirm that rBipA was electrophoresed and transferred to membranes (Fig. 6D to F). Collectively, these data further support that rBipA can distinguish between LD and TBRF spirochete infections.

**TABLE 4:** Sera reactivity to rBipAs^*a*^

Sample	ELISA			Immunoblot			Origin and reference
	*Bt*	*Bp*	*Bh*	*Bt*	*Bp*	*Bh*	
TBRF-neuroborreliosis	+	–	–	+	–	–	Patient from Austin, TX (25)
Negative human serum	–	–	–	–	–	–	Laboratory sample
CDC-74	–	–	–	–	–	–	LD positive (NL) (36)
CDC-76	–	–	–	–	–	–	LD positive (EL/A) (36)
CDC-78	–	–	–	–	–	–	LD positive (EL/A) (36)
CDC-79	–	–	–	–	–	–	LD positive (LA) (36)
CDC-85	–	–	–	–	–	–	LD positive (LA) (36)
CDC-86	+	+	–	–	–	–	LD positive (LA) (36)
CDC-87	–	–	–	–	–	–	LD negative (MN) (36)
CDC-89	–	–	–	–	–	–	LD positive (EL/C) (36)

a *Bt*, *B. turicatae*; *Bp*, *B. parkeri*; *Bh*, B. hermsii; +, reactive; –, not reactive; NL, NL, neuro Lyme; EL/A, early Lyme/acute; LA, Lyme arthritis; MN, mononucleosis; EL/C, early Lyme/convalescent.

## DISCUSSION

With the need for improved diagnostic tests for TBRF spirochetes, the objective of this study was to evaluate immunological responses toward rBipA from North American species of TBRF spirochetes in different vertebrate hosts. Our study focused on species transmitted by argasid ticks because the ticks are elusive rapid feeders and are rarely found attached to the host. This is compared to Ixodes scapularis, a vector of the hard tick-borne relapsing fever-causing Borrelia miyamotoi, which feeds for days. Consequently, individuals more often recover I. scapularis, and testing can be performed on the tick to evaluate whether it is infected with B. miyamotoi. Individuals fed on by argasid ticks rarely know they have been exposed, further hindering an accurate diagnosis. While prior work indicated the antigenicity of BipA in B. hermsii and *B. turicatae*, this study demonstrated that the protein is produced and antigenic in *B. parkeri* as well.

Given the nuances of TBRF borreliosis between species, identifying the causative agent can aid in treatment in regions where different species overlap. For example, the vectors of B. hermsii, *B. turicatae*, and *B. parkeri* are distributed in states throughout the Southwest (11). One significant difference in the manifestation of the disease between the three species is that *B. turicatae* is neurotropic. Tetracycline antibiotics that are typically prescribed for patients with TBRF fail to cross the blood-brain barrier (2). Ceftriaxone, a cephalosporin antibiotic, is the treatment of choice during infection with neuroborreliosis caused by *B. turicatae* (42). Detecting early antibody responses to rBipA could aid in determining the species causing infection. While in our study we did not differentiate between acute and convalescent antibody responses generated toward rBipA, IgM detection in acute-phase serum samples were previously demonstrated using the TBRF spirochete antigen, GlpQ (43). In that study, acute-phase serum samples were collected from patients during early infection when spirochetes were first visualized in the blood. While these samples were initially determined to be IgG negative for GlpQ (44), in subsequent work they were confirmed as IgM-positive (43). This suggested that detecting an IgM response could aid in diagnosing early infection. Similar studies will evaluate IgM responses toward rBipA to determine whether acute-phase serum samples can differentiate between the species causing infection.

An aim of this study was to establish criteria to consider in the development of assays for TBRF spirochetes in clinical and field settings. For example, serum dilutions between 1:200 and 1:500 established a species-specific cutoff for the rodent samples evaluated. For canines, the species-specific dilution cutoffs were 1:600. Since domesticated canines are susceptible hosts to species of TBRF spirochete (45–48), these findings are relevant in regions where *B. turicatae*, *B. parkeri*, and B. hermsii may overlap. For nonhuman primate serum samples, the species-specific dilution cutoffs were 1:3,200. However, with these serum samples, we only observed a 2-fold difference in reactivity to *B. turicatae* and *B. parkeri* rBipA. While human serum samples were not available for this study, these findings suggest that the antibody responses generated in high-order mammals may bind to BipA epitopes conserved between the two species. To improve the diagnostic capability of BipA, additional work should focus on the identification of species-specific epitopes.

Continual assessment of the sensitivity and specificity of rBipA will need to occur with the emergence and identification of novel relapsing fever *Borrelia* species. Recent findings indicate that three additional TBRF *Borrelia* species are likely endemic to North America, *B. mazzottii*, “*Candidatus* B. johnsonii,” and B. miyamotoi. While no laboratory isolates of *B. mazzottii* and “*Candidatus* B. johnsonii” exist, each has been identified in vectors endemic to North America and has been associated with human disease (15, 16, 49). *B. mazzottii* was identified in Ornithodoros talaje from Northern Mexico (16), and “*Candidatus* B. johnsonii” was detected in Carios kelleyi (bat ticks) from Iowa (49). B. miyamotoi, the TBRF *Borrelia* species transmitted by species of *Ixodes* ticks (50), has been implicated in human diseases in the western, upper midwestern, and northeastern United States (51–53). As animal models are developed and positive serum samples obtained from vertebrate hosts, studies can be implemented to assess the specificity of rBipA from each of these *Borrelia* species.

Our findings also supported that rBipA can be used to distinguish between infections caused by LD and TBRF spirochetes. We showed that serum from patients with early and disseminated LD (Lyme arthritis and neurological disease) do not cross-react with rBipA from *B. turicatae*, *B. parkeri*, and B. hermsii. This is important in regions where Lyme borreliosis is being diagnosed but epidemiological support is absent (54). For example, there is growing evidence of neuroborreliosis disease in Mexico that is repeatedly attributed to LD, and TBRF is rarely considered. Two large studies diagnosed patients with neurological symptoms as having LD based on serological assays (55, 56). In the first study, 27% (168/606) of patients with cranial neuritis, radiculoneuritis, meningitis, or encephalomyelitis were diagnosed with LD (55). The second study reported that 13% (25/191) of patients with facial palsy were seropositive for LD-causing spirochetes (56). In both studies, the serological approach used whole-spirochete lysates from LD bacteria, and serological cross-reactivity to TBRF spirochetes was not considered despite the pathogens being endemic to the region and presenting with similar neurological symptoms (9, 54, 57). Additionally, since serum from TBRF-positive patients can cross-react with Lyme serological assays (10, 26, 58), TBRF should be considered in addition to LD.

A limitation of this study was the use of laboratory mice for serum sample assessment and the lack of human TBRF samples. We reasoned that assessing laboratory mice was the first step to determine whether rBipA was a species-specific antigen, and future studies will continue to assess the antigenicity of BipA from natural vertebrate hosts that maintain the disease. Furthermore, obtaining serum samples from human patients confirmed to be infected with TBRF spirochetes is challenging because of a lack of awareness in the medical community, and confirmed cases are often retrospective. However, a population that should be investigated is those who experience homelessness in the southern United States. For example, in Texas shelters have been established in locations where we have recovered infected ticks (59). However, exposure frequencies in this population are unknown.

TBRF is a neglected vector-borne disease, and our findings continue to support the diagnostic potential of BipA. Without a standard serological test that accurately determines the species of the causative agent of TBRF, the misdiagnosis and underreporting of TBRF continues to be a possibility. The use of rBipA in serological assays can aid in determining the *Borrelia* causing disease in Mexico and other regions of North America. Furthermore, the antigen will help elucidate our understanding of the epidemiology, species-specific disease manifestations, and emergence of new endemic foci of TBRF spirochetes.

## MATERIALS AND METHODS

### Ethics statement.

The Institutional Care and Use Committee (IACUC) at Baylor College of Medicine approved studies performed using mice, with protocol numbers AN-6563 and AN-6580. Their laboratory animal program complies with standards and guidance that were established by the Association for Assessment and Accreditation of Laboratory Animal Care and the National Institution of Health of Laboratory Animal Welfare. The veterinary staff provided animal care and husbandry.

### Illumina sequencing.

Genomic DNA was isolated from 40-mL cultures of *B. parkeri* SLO and B. hermsii HCT-4 by phenol/chloroform extraction as described previously (60). Genomic DNA was sent to the Microbial Genome Sequencing Center (MiGS Center, Pittsburgh, PA, USA) for Illumina sequencing. The DNA was prepared using the Illumina Nextera 2 × 150-bp library prep kit and sequenced on a NextSeq 550 instrument. Base-called FASTQ files were delivered by the MiGS Center to us, and these files were processed with fastp (v0.20.0) (61). The paired-end data were quality score-filtered and corrected using the -q 20 and -c options, respectively. The sequencing data were assembled using the SPAdes assembler (v3.13.1) (62). An assembly of *B. parkeri* SLO and B. hermsii HCT-4 scaffolds was generated, and these scaffolds were searched via blastn using *B. parkeri* HR1 BipA (AHF45615.1) and B. hermsii DAH BipA (ADF49584.1) as a query and the scaffolds as a subject, respectively. All computational work conducted was completed on a Thelio Massive system with an Intel Xeon Gold 6230 processor and 126 Gb ECC DDR4 2933 MHz RAM (System 76, Denver, CO, USA). The amino acid sequences of the mature BipA proteins from *B. turicatae* 91E135 (YP_008145285.1), *B. parkeri* HR1 (AHF45615.1), *B. parkeri* SLO (MW589542), B. hermsii DAH (ACS27065.1), and B. hermsii HCT-4 (MW589543) were aligned using ClustalW (https://www.genome.jp/tools-bin/clustalw).

### Production of *B. turicatae*, *B. parkeri*, and B. hermsii rBipA.

The *bipA* gene from *B. turicatae* 91E135, *B. parkeri* HR1, and B. hermsii DAH was codon-optimized using GenScript and expressed from the pET-19b plasmid as 10× histidine-tagged fusion proteins. The pET-19b plasmids containing *bipA* genes were transformed into Escherichia coli BL21(DE3) chemicompetent cells (Invitrogen by Thermo Fisher Scientific, Waltham, MA, USA), and protein production was induced following the manufacturer’s instructions. Cells were pelleted at 5,000 × *g* for 10 min, resuspended in wash buffer (50 mM Tris-HCl, 100 mM NaCl, 1 Mm EDTA, 0.5% Triton X-100, 1 mM DTT; pH 8.0) with 1× EDTA-free protease inhibitor cocktail (cOmplete; Roche, Mannheim, Germany), and lysed via sonication for 3 min at 75% power (20 s on, 10 s off). Cellular lysates were spun at 15,000 × *g* for 10 min at 4°C and washed and lysed three more times. The pellet was suspended in 10 mL of 1× binding buffer (0.5 M NaCl, 20 mM Tris-HCl, 5 mM imidazole; pH 8.0) with 4 M urea overnight at 4°C. An additional 10 mL of 1× binding buffer with 4 M urea was added, and lysates were rocked at room temperature for 1 h to dissolve the pellet. The lysates were centrifuged at 15,000 × *g* for 15 min at room temperature. Supernatants were filtered through a 0.45-μm filter, and rBipA was purified using 5 mL HisTrap FastFlow columns (GE Healthcare, Uppsala, Sweden) following the manufacturer’s instructions. rBipA proteins were concentrated, and urea was removed through diafiltration using Amicon Ultra centrifugal filters (Millipore Sigma, Burlington, MA, USA) following the manufacturer’s instructions. Purity was confirmed using NanoDrop and SDS-PAGE with SimplyBlue SafeStain (Invitrogen, Carlsbad, CA, USA) staining per the manufacturer’s microwave protocol. Protein concentrations were determined with the Bradford assay (Bio-Rad, Hercules, CA, USA).

### Bacterial strains and generation of protein lysates.

Low-passaged (≤10 laboratory passages) cultures of B. burgdorferi B31 A3 (63), *B. turicatae* 91E135 (64), *B. parkeri* SLO, and B. hermsii HCT-4 (29) were grown at 35 to 37°C to densities of  >1 × 10^7^ cells mL^−1^ in 40 mL modified Barbour-Stonner-Kelly (mBSK) medium with 12% rabbit serum (65, 66). Cultures were centrifuged at 11,000 × *g* for 20 min at 10°C. Cells were washed twice with phosphate-buffered saline (PBS) plus 5mM MgCl_2_ and centrifuged following each wash. Cells were resuspended in a 1:1 solution of PBS plus 5 mM MgCl_2_:2× Laemmli sample buffer (SB) (Bio-Rad, Hercules, CA, USA) with 2-mercaptoethanol (BME) (Sigma, St. Louis, MO, USA) at a density of 2 × 10^6^ cells μL^−1^.

### Producing antisera to recombinant BipA (rBipA).

Rabbit anti-rBipA immune serum was generated by Cocalico Biologicals, Inc. (Reamstown, PA, USA) as described previously (67). Two rabbits were immunized twice with 50 μg of rBipA via intraperitoneal injections using complete Freund’s adjuvant. At 2-week intervals, three subsequent immunizations were performed using incomplete Freund’s adjuvant. Serum samples were collected and tested for specificity to BipA using *B. turicatae* Δ*bipA* mutants.

### Generation of *B. turicatae* Δ*bipA* mutants.

*B. turicatae* Δ*bipA* mutants were generated as previously reported (67). Primers Btur F primer–1000 and Btur R primer+1000 (Table 5) amplified the *bipA* gene along with 1,000 bp up- and downstream of the gene (Fig. S1A). The amplicon was cloned into the pCR-XL-TOPO plasmid and transformed into Top10 E. coli (Life Technologies, Carlsbad, CA, USA) (Fig. S1B). To remove the *bipA* gene from the pCR-XL-TOPO plasmid, primers Btur F-Del*Nhe*I and Btur R-Del*Avr*II (Table 5) were used to PCR amplify the construct and insert NheI and AvrII restriction sites (Fig. S1C). The amplicon was then doubly digested (Fig. S1D). To form the deletion construct; P_flgB_-gent was amplified from pBhSV-2::Bt P_flgB_-gent (67) using Btur-flgBgent*Avr*II and *gentR-*SpeI (Table 5) and cloned into the pCR-XL-TOPO plasmid containing the *bipA* flanking DNA (Fig. S1E). Transformation of the deletion construct into *B. turicatae* 91E135 was performed as previously described (67). PCR was used to confirm the deletion of bipA in two clones (Fig. 2). The clones were used to test the specificity of the rabbit anti-rBipA immune serum (Fig. 2).

**TABLE 5:** Oligonucleotides used in this study

Primer name	Sequence (5′–3′)^*a*^
Btur F primer–1000	GTAGGTGATTTATTTGTTGATGGCATTATG
Btur R primer+1000	ATCTTGATCTACCATTAATCTTAATAGCACTCC
Btur R-Del*AvrII*	**TCCTAGG**CACACAAAATATTAAGATAATAATATAGCAATAAAATTGA
Btur F-Del*NheI*	**TGCTAGC**AGCTACAAATTAATGTAATGATTTAAGAATTTACTCTAAG
BturF-flgBgent*AvrII*	**TCCTAGG**AGCACCCGGTAGCAAGTTAAAAAAATTTGAAATAAACTTG
*gent* R-*SpeI*	AA**ACTAGT**CTCGGCTTGAACGAATTGTTAGG
flaLL	ACATATTCAGATGCAGACAGAGGT
flaRL	GCAATCATAGCCATTGCAGATTGT
Bt bipA For D-TOPO	CACCATGTGGTTTGTAAGGAGGGTGGATAT
Btur bipA R2	AATTGAATTTATTGAATTTTCATTTTCTGTT
Gent 3′	AAACTAGTCTCGGCTTGAACGAATTGTTAGG
G2	CAAAGTTAGGTGGCTCAAGTATGG

a Bold nucleotides indicate restriction enzyme site.

### Generation of infected ticks and mammalian serum samples.

Moreover, to obtain additional infected cohorts of ticks used to infect mice, Institute of Cancer Research (ICR) mice were infected with 1 × 10^6^ cells of *B. turicatae* or *B. parkeri* by needle inoculation. When these animals were spirochetemic (~1 × 10^6^ spirochetes mL^−1^), cohorts of ticks were allowed to engorge. Ticks were housed at ~85% relative humidity on a 12-h light cycle. After molting, they were fed on naive mice, and transmission was assessed by daily sampling of blood from tail nicks.

The human serum samples from LD patients originated from the CDC (36, 68), and the human TBRF-positive serum sample was derived from a patient from Austin, TX (25). LD patient serum samples came from individuals with early or disseminated (Lyme arthritis or neurological) LD (36). Early LD patients were confirmed by clinical diagnosis, PCR, or isolation of spirochetes (68). The human serum sample origins and descriptions are summarized in Table 4. Domestic dog, rhesus macaque, and murine serum samples originated from prior animal studies (27, 34, 35, 41), and Table 1 summarizes the origin of serum samples used in this study. To infect mice with B. hermsii, sixteen female ICR mice were inoculated by intraperitoneal needle injection with ~1 × 10^7^ cells of the HCT-4 isolate (29). Mice were monitored daily for infection and exsanguinated 17 to 31 days postinoculation. Serum was extracted from whole blood following centrifugation at 4,200 × *g* for 15 min and stored at 4°C.

### Proteinase K assays.

Proteinase K assays were performed to determine surface localization of BipA in *B. turicatae* and *B. parkeri* as described previously (35). Cells were incubated with 0, 5, 50, or 200 μg mL^−1^ of proteinase K (Promega, Madison, WI, USA) for 15 min at room temperature. PBS plus 5 mM MgCl_2_ was used as the vehicle (0 μg mL^−1^) control. Proteinase K was inactivated by boiling samples at 100°C for 10 min. Samples were mixed with a 1:1 ratio of 2× SB plus BME, and Western blots were performed for BipA and FlaB as described above.

### Serologic assays.

Proteins from whole-cell lysates (1 × 10^7^ cells) and 1 μg of rBipA from *B. turicatae*, *B. parkeri*, and B. hermsii were separated by SDS-PAGE using Any kD Mini-PROTEAN TGX precast protein gels (Bio-Rad) at 80 V for 90 min. Proteins were transferred to Immobilon polyvinylidene difluoride (PVDF) membranes (Merck Millipore, Carrigtwohill, County Cork, Ireland) at 100 V for 60 min. Blots were probed for BipA using polyclonal rabbit anti-*B. turicatae* rBipA (anti-*Bt-*rBipA) antibodies at a 1:200 dilution, for FlaB using chicken anti-*B. turicatae* rFlaB (anti-*Bt-*rFlaB) at a 1:200 dilution (67), or with murine serum or human serum at a 1:200 dilution. Horseradish peroxidase recombinant (HRP-rec) protein G (Invitrogen, Rockford, IL, USA), anti-chicken IgG (Rockland, Gilbertville, PA, USA), or anti-human IgA/G/M-HRP (Millipore, Temecula, CA, USA) were used as secondary antibodies at a 1:4,000 dilution. Blots were developed and analyzed with ChemiDoc MP (Bio-Rad, Hercules, CA, USA) and ImageLab (Bio-Rad), respectively. Relative optical densities were determined for each rBipA band compared to the rBipA from other species of relapsing fever *Borrelia*. Following development, blots were reprobed with monoclonal anti-polyhistidine-peroxidase antibody (Sigma, St. Louis, MO, USA) at a 1:4,000 dilution to detect recombinant protein. For the uninfected control serum samples, the relative optical densities were compared based on rBipA reactivity in uninfected control immunoblots that were reprobed with anti-polyhistidine-peroxidase antibody.

For ELISAs, polystyrene plates were coated with 100 ng of rBipA from each TBRF species. Diluent (PBS plus 5% horse serum plus 0.1% Tween 20 plus 0.001% dextran sulfate) was used to block the wells of each plate for 2 h at room temperature. Wells were probed with serum samples (mouse samples diluted 1:500, canine and NHP samples diluted in 1:1 serial dilutions starting at 1:200) for 1 h at room temperature. Plates were washed (1× PBS plus 0.1% Tween 20) and probed with HRP-rec protein A (Invitrogen, Rockford, IL, USA) at a 1:4,000 dilution for 1 h at room temperature. Plates were washed again and incubated with HRP substrate (Sera Care, Milford, MA, USA) for 30 min. The absorbance of each well was measured at 405 nm. Each serum sample was run in triplicate on each plate. Serum samples from uninfected animals (at least three animals of each species) were used as negative controls. Analysis of variance (ANOVA) statistical analysis of data was performed using GraphPad Prism 8 (* = *P ≤ *0.01). We considered any serum sample positive if it had an absorbance greater than the mean plus three times the standard deviation of the negative controls, as previously reported (43). The sensitivity of each rBipA was calculated as follows:

$$\frac{\text{no.}\;\text{of}\;\text{true}\;\text{positives}}{\text{no.}\;\text{of}\;\text{true}\;\text{positives}\;+\;\text{no.}\;\text{of}\;\text{false}\;\text{negatives}}$$

The specificity of each rBipA was calculated as follows:

$$\frac{\text{no.}\;\text{of}\;\text{true}\;\text{negatives}}{\text{no.}\;\text{of}\;\text{true}\;\text{negatives}\;+\;\text{no.}\;\text{of}\;\text{false}\;\text{positives}}$$

The specificity and sensitivity rates were calculated based on ELISAs that were repeated twice.
