# Supplementary material for: Characterization of Immunological Responses to Borrelia Immunogenic Protein A (BipA), a Species-Specific Antigen for North American Tick-Borne Relapsing Fever
Source: Microbiol Spectr. 2022 May 17;10(3):e01722-21. doi: 10.1128/spectrum.01722-21 (PMC9241729; doi:10.1128/spectrum.01722-21)
Supplement: SUPPLEMENTAL FILE 1 — Supplemental material. Download spectrum.01722-21-s0001.pdf, PDF file, 0.3 MB [file spectrum.01722-21-s0001.pdf]

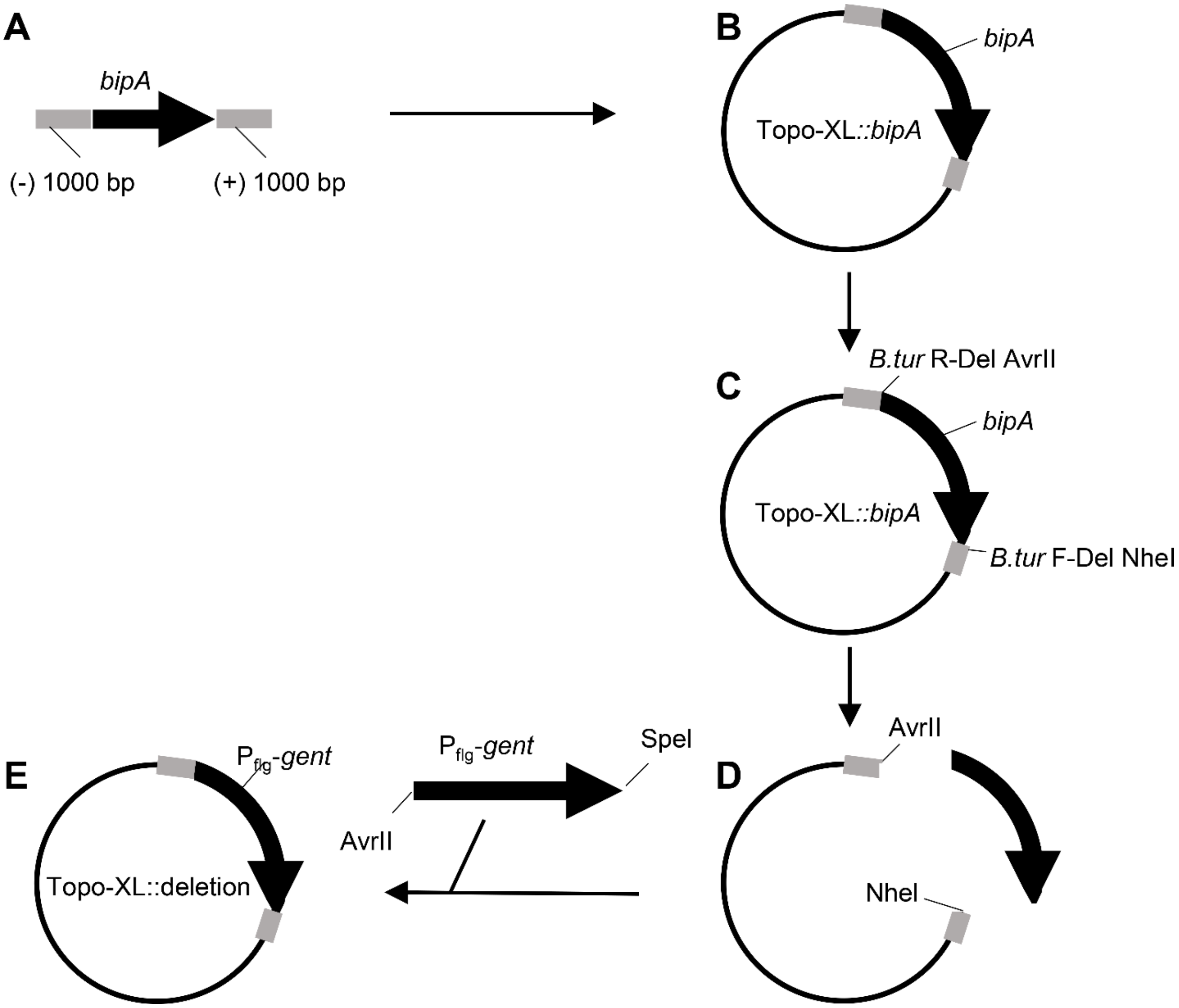

Sup. Fig. 1: *Borrelia turicatae bipA* deletion vector construct. *B. turicatae bipA* gene ± 1000bp (upstream and downstream) was amplified (A) and cloned into the Topo-XL vector creating the Topo-XL::*bipA* construct (B). Through PCR amplification, which added *AvrII* and *NheI* restriction sites, and subsequent double digestion, *bipA* was removed (C and D). Adding *AvrII* and *SpeI* restriction sites to *flgBp-gent* through PCR amplification, double digesting, and ligating, generated the knockout vector (E).

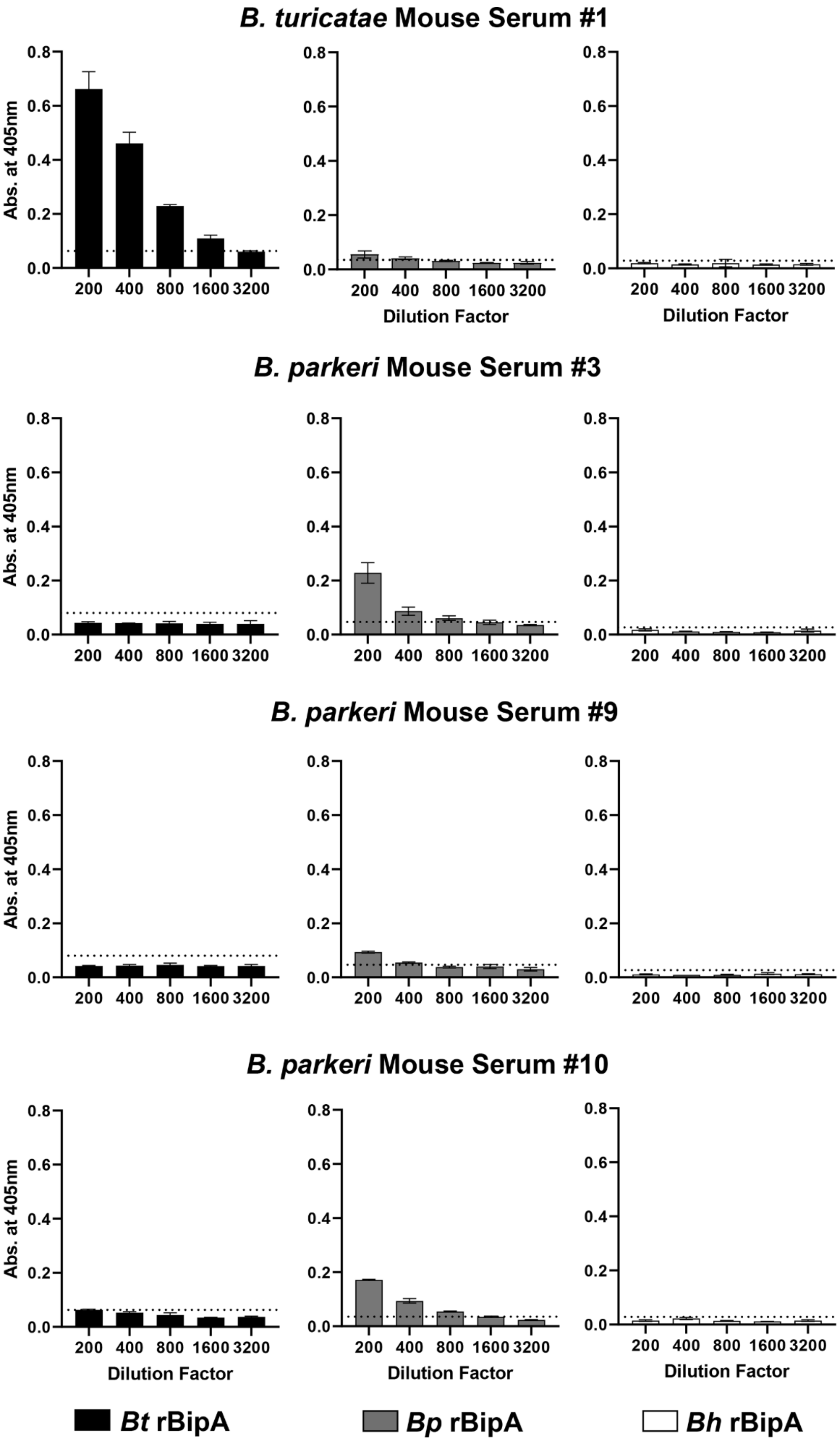
B. turicatae Mouse Serum #1
B. parkeri Mouse Serum #3
B. parkeri Mouse Serum #9
B. parkeri Mouse Serum #10
Abs. at 405nm
0.0
0.2
0.4
0.6
0.8
200
400
800
1600
3200
Dilution Factor
Bt rBipA
Bp rBipA
Bh rBipA

Sup. Fig. 2: Antibody titers from mice that reacted to rBipA from more than one TBRF spirochete species. Shown are representative antibody titers. The dashed line (….) depicts the threshold of a positive result for each rBipA (mean + three times the standard deviation of uninfected controls).
